# FLIP(L): the pseudo‐caspase

**DOI:** 10.1111/febs.15260

**Published:** 2020-03-12

**Authors:** Peter Smyth, Tamas Sessler, Christopher J. Scott, Daniel B. Longley

**Affiliations:** ^1^ The Patrick G Johnston Centre for Cancer Research Queen’s University Belfast UK

**Keywords:** apoptosis, autophagy, caspase, DISC, FLIP(L), necroptosis, pseudo-caspase, pseudoenzymes

## Abstract

Possessing structural homology with their active enzyme counterparts but lacking catalytic activity, pseudoenzymes have been identified for all major enzyme groups. Caspases are a family of cysteine‐dependent aspartate‐directed proteases that play essential roles in regulating cell death and inflammation. Here, we discuss the only human pseudo‐caspase, FLIP(L), a paralog of the apoptosis‐initiating caspases, caspase‐8 and caspase‐10. FLIP(L) has been shown to play a key role in regulating the processing and activity of caspase‐8, thereby modulating apoptotic signaling mediated by death receptors (such as TRAIL‐R1/R2), TNF receptor‐1 (TNFR1), and Toll‐like receptors. In this review, these canonical roles of FLIP(L) are discussed. Additionally, a range of nonclassical pseudoenzyme roles are described, in which FLIP(L) functions independently of caspase‐8. These nonclassical pseudoenzyme functions enable FLIP(L) to play key roles in the regulation of a wide range of biological processes beyond its canonical roles as a modulator of cell death.

## Graphical abstract

FLIP(L) is to date the only true pseudo‐caspase identified in the human proteome. As a classical pseudoenzyme, it functions as a regulator of its active homologs, the apoptosis‐initiating caspases, caspase‐8 and caspase‐10. Additionally, FLIP(L) functions independently of these caspases. These nonclassical pseudoenzyme functions enable FLIP(L) to play key roles in the regulation of a wide range of biological processes beyond its canonical roles as a modulator of cell death.

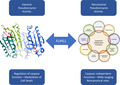

**Abbreviations**
AIM2: interferon‐inducible protein AIM2
AKT: protein kinase B
APC: adenomatous polyposis coli
ASC: apoptosis‐associated speck‐like protein containing CARD
ATG: autophagy‐related genes
Bcl‐2: B‐cell lymphoma 2
BCLAF1: Bcl‐2 associated transcription factor 1
Bid: BH3‐interacting domain death agonist
CD95: cluster of differentiation 95
DAMPs: damage‐associated molecular pattern
DD: death domain
DED: death effector domain
DR: death receptor
ERK: extracellular signal‐regulated kinase
FADD: FAS‐associated death domain protein
HIN: hematopoietic interferon‐inducible nuclear antigens
IFN: interferon
IKK: IκB kinase
IL: interleukin
IRF3: interferon regulatory transcription factor 3
ITF2: immunoglobulin transcription factor‐2
JNK: c‐Jun N‐terminal kinase
LC3: microtubule‐associated protein 1A/1B‐light chain 3
MAP: mitogen‐activated protein
MEKK1: MAP/ERK kinase kinase 1
MKK7: MAP kinase kinase 7
MLKL: mixed lineage kinase domain‐like protein
NES: nuclear export signal
NF‐κB: nuclear factor kappa‐light‐chain‐enhancer of activated B cells
NGF: nerve growth factor
NLRP3: NACHT, LRR, and PYD domains‐containing protein 3
NLS: nuclear localization sequence
PERK: protein kinase RNA‐like endoplasmic reticulum kinase
PYD: pyrin domain
RAF‐1: RAF proto‐oncogene serine/threonine‐protein kinase
RIPK1: receptor‐interacting serine/threonine‐protein kinase 1
SGLT1: sodium/glucose cotransporter 1
TIP49: TBP‐interacting protein 49
TLRs: Toll‐like receptor
TNF: tumor necrosis factor
TNFR: tumor necrosis factor receptor
TRADD: TNFR1‐associated death domain
TRAF1: TNF receptor‐associated factor
TRAIL: TNF‐related apoptosis‐inducing ligand
Wnt: Wingless‐related integration site

## Introduction

Initially identified over 50 years ago, pseudoenzymes have recently been the subject of increased interest as the originally unanticipated extent of their biological importance has become apparent 1. Pseudoenzymes can be defined as noncatalytic homologs of active enzymes. As such, they possess considerable sequence similarity to their catalytically active counterparts, but crucially, changes to key amino acid residues render them incapable of catalysis 2. While initially considered to act solely as regulators of their active homologs, the diverse range of functions that these proteins have subsequently been shown to be involved in and their mechanistic diversity highlights their frequent importance beyond the regulation of their closely related active counterparts 3, 4, 5.

It is thought most likely that pseudoenzymes evolved as a result of gene duplication events, with one of the duplicated genes no longer having the selective pressure of maintaining an active catalytic site 6. This classic pattern of duplication, redundancy, and development of functional novelty is at the heart of evolution. As the functional roles of pseudoenzymes are often linked to the function of the active enzyme, it has been hypothesized that pseudoenzyme functions are often preexisting functions of the catalytically active precursor, which then became the primary function of the pseudoenzyme 4. However, this is not always the case, and some pseudoenzymes have functions that are quite distinct from their active counterpart. As such, the biological functions of pseudoenzymes are broad and range from allosteric binding of their active homolog, occlusion, and sequestering of the active enzyme’s substrate through to more diverse regulation of protein localization, processing, and trafficking 1.

It is estimated that pseudoenzymes comprise ~ 10% of proteomes, a further indication of their biological importance 1. Pseudoenzymes can be broken down into distinct subclasses, such as pseudokinases and pseudoproteases 2, 7, 8. In this review, we will discuss the long splice form of c‐FLIP (cellular Fas‐associated protein with death domain (FADD)‐like interleukin‐1β‐converting enzyme‐inhibitory protein), FLIP(L), the only true pseudo‐caspase so far identified in the human proteome.

## c‐FLIP

**Fig. 1:**
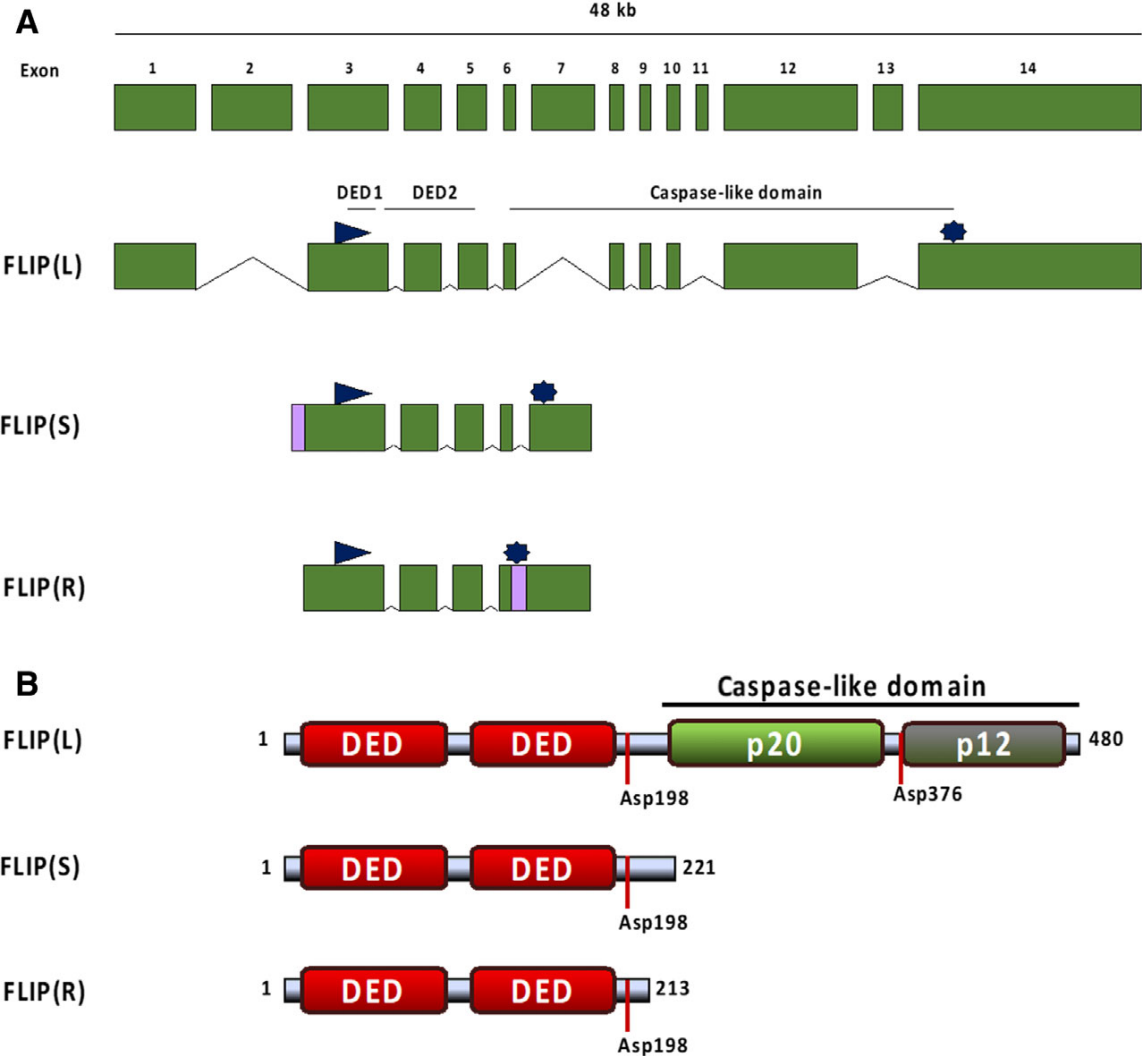
Schematic representation of cellular FLIP splice variants. (A) The genomic organization of the splice variants of CFLAR/c‐FLIP. The 48‐kb CFLAR gene is comprised of 14 exons. While transcribed into 11 splice forms, only FLIP(L), FLIP(S), and FLIP(R) are expressed at the protein level. Translation start and stop sites are indicated by triangles and stars, respectively. 111(B) All splice variants contain tandem DEDs allowing recruitment to the DISC. Only FLIP(L) is a true pseudo‐caspase, possessing a pseudoenzymatic region that can be cleaved by DISC‐bound procaspase‐8 at Asp 376 to form p43‐ and p12‐FLIP(L) cleavage products. The FLIP(L) pseudo‐caspase domain also contains a NLS and NES. FLIP (S/R) lack the pseudo‐caspase domain of FLIP(L). They differ from each other in their C terminus.

Encoded by the *CFLAR* gene (located on chromosome 2q33.1), c‐FLIP has been shown to be involved in regulating a number of key cellular functions, including apoptosis, necroptosis, and autophagy. c‐FLIP is expressed in humans as a number of splice variants, namely FLIP(L) (FLIP long), FLIP(S) (FLIP short), and FLIP(R) (FLIP Raji). Each of these splice variants possesses two tandem N‐terminal protein–protein interaction domains termed death effector domains (DEDs). These DEDs facilitate homotypic interactions with other proteins bearing DEDs, most importantly procaspase‐8 and procaspase‐10 and FADD. While each possesses tandem N‐terminal DEDs, c‐FLIP splice forms differ at their C terminus 9, 10, 11, 12, 13 (Fig. 1):

- FLIP(S) is a 26‐kDa protein comprised of two DEDs followed by a 20 amino acid unstructured sequence, which plays an important role in its ubiquitination and subsequent proteasomal degradation.
- FLIP(R) is a 24‐kDa protein notable for its restricted expression pattern, being found mainly in T cells. Like FLIP(S), it is comprised of two DEDs, although its C‐terminal region is different due to alternative splicing.
- FLIP(L) is a 55‐kDa protein, the C terminus of which possesses a region which is highly similar to the equivalent regions of procaspase‐8 and procaspase‐10. FLIP(L) differs from these caspases; however, in that this region is catalytically inactive hence marking it as a classic pseudoprotease.

### Evolutionary conservation

The *CFLAR* gene is located within the same chromosomal region (2q33.1) as the genes encoding its paralogs caspase‐8 (*CASP8*) and caspase‐10 (*CASP10*) 14, 15, 16. This proximity, coupled with the similarities in the encoded proteins (two DEDs with caspase/pseudo‐caspase domains), indicates that these genes are derived from a common ancestral caspase gene, with divergence following two gene duplication events 17. Subsequent construction of molecular phylogenetic trees for *CFLAR* and its paralogs has shed further light on its evolutionary origins. Across a variety of species, including humans, it is apparent that *CFLAR* has a unique and independent evolutionary path, which is distinct from those of *CASP8* and *CASP10*. Furthermore, it was noted that in humans, the molecular evolutionary rate of *CFLAR* is 1.7× faster than that of *CASP8* 18. This relatively rapid rate of molecular change postdivergence from its ancestral caspase is consistent with c‐FLIP’s (particularly FLIP(L)’s) multiple functions that extend beyond those involving caspases‐8/10.

An interesting point of note in the evolution of *CFLAR*, *CASP8*, and *CASP10* is seen in rodents. While *CASP8* and *CASP10* are mostly conserved throughout vertebrate evolution, *CASP10* is not present in a subset of rodents. While present in the guinea pig and squirrel, this gene is absent in both the rat and the mouse 16. It is thought that this loss of the *CASP10* gene occurred in and around the cretaceous period, when the divergence of these rodent clades is thought to have taken place 16, 19.

**Fig. 2:**
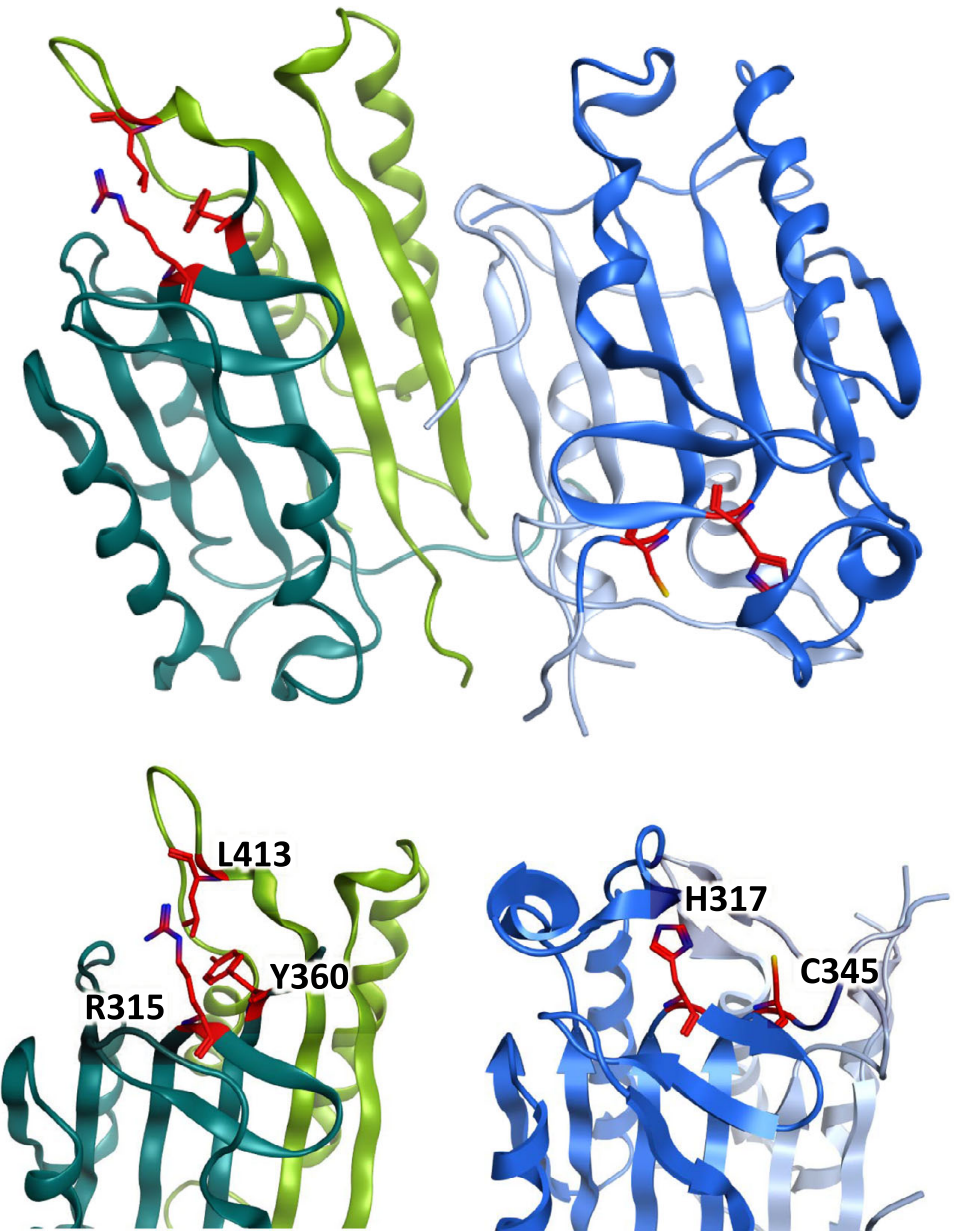
(Upper) Representation of the crystal structure of the caspase‐8 and FLIP(L) heterodimer (PDB 3H13) 33. The formation of the heterodimer between the (pseudo)catalytic domains of FLIP(L) (green) and caspase‐8 (blue) is energetically favorable compared with the caspase‐8 homodimer. This explains why FLIP(L) can promote caspase‐8 activation and apoptosis in certain conditions (see text for details), as when part of a FLIP(L)/caspase‐8 heterodimer, caspase‐8 has restricted enzymatic activity that is capable of efficiently cleaving and processing adjacent procaspase‐8 homodimers, the rate‐limiting step of procaspase‐8 activation. (Lower) The amino acid residues in the active caspase domain of caspase‐8 and the equivalent pseudo‐caspase region of FLIP(L) have been highlighted in red. Notably, the amino acid changes in FLIP(L)’s pseudo‐caspase domain that render it catalytically inactive are evolutionarily conserved, suggestive of important functionality.

At the protein level, this evolutionary divergence manifests itself most obviously in the pseudo‐caspase domain of FLIP(L), where amino acid substitutions have resulted in loss of catalytic activity. In both caspase‐8 and caspase‐10, the catalytic activity is imparted by a catalytic dyad composed of histidine and cysteine residues. In comparison, in FLIP(L), these residues are absent, being replaced by arginine (R) and tyrosine (Y) residues (R^315^ and Y^360^ in humans), respectively. Comparative analysis of the pseudo‐caspase domain in FLIP(L) has further identified the importance of a leucine residue (L^413^ in humans). Conserved across vertebrates, this leucine is able to form a distinct tertiary structure triad with the aforementioned conserved arginine and tyrosine residues 13, 18, 20 (Fig. 2).

## FLIP(L)’s canonical functions

The biological roles of FLIP(L) can be roughly subdivided into those in which FLIP(L) forms complexes via its tandem DEDs with FADD and procaspase‐8 and/or procaspase‐10 and those where it acts independently of FADD and its genetic paralogs.

### The death‐inducing signaling complex, DISC

One of the most fundamental and widely studied roles of FLIP(L) is its modulation of signaling of tumor necrosis factor (TNF) receptor superfamily members TNF‐related apoptosis‐inducing ligand R1 (TRAIL‐R1)/death receptor 4 (DR4), TRAIL‐R2/DR5, and Fas/cluster of differentiation 95 (CD95) within the extrinsic apoptotic pathway 21, 22, 23. Activation by their respective ligands TRAIL or FasL (CD95L), which are mainly expressed on the surface of immune effector cells, results in receptor clustering and initiates the formation of the death‐inducing signaling complex (DISC). DISC formation occurs in a stepwise manner: Initially, the adaptor protein FADD is recruited via its death domain (DD), which interacts with the DD of the activated receptor via homotypic interactions. This interaction enables FADD’s N‐terminal DED to form homotypic interactions with other DED‐containing proteins, such as the pro‐forms of caspase‐8 and caspase‐10 (procaspase‐8 and procaspase‐10).

**Fig. 3:**
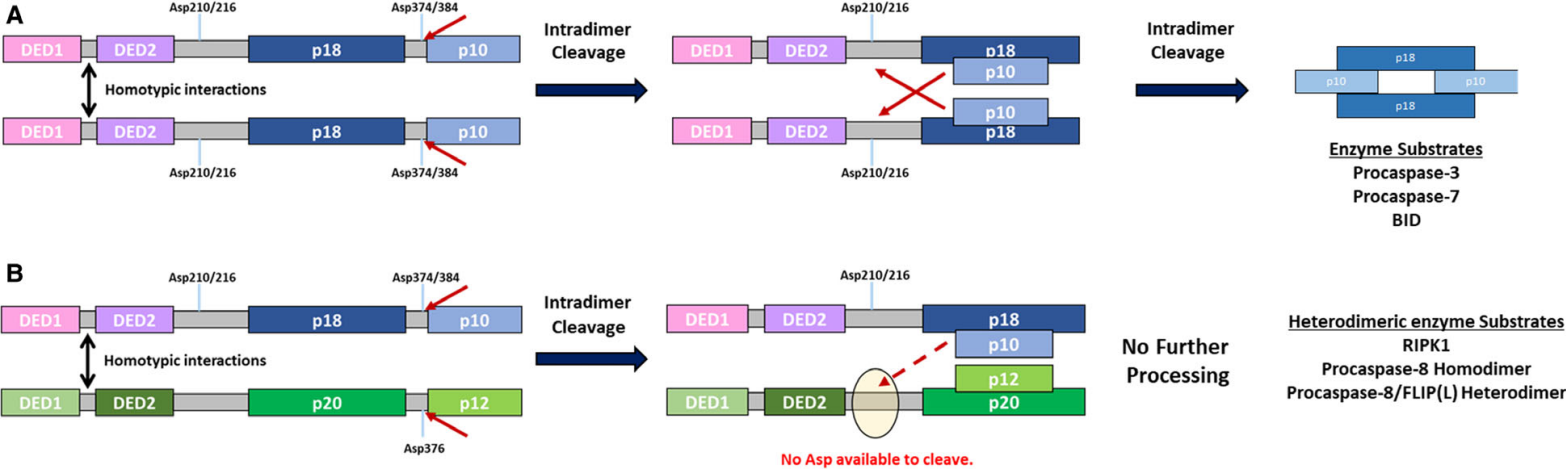
Processing and substrates of procaspase‐8 homodimers and procaspase‐8/FLIP(L) heterodimers. (A) Homodimerization is initially mediated via homotypic DED interactions. Within a homodimer, the caspase‐8 catalytic domains are arranged in an antiparallel fashion. This creates an enzymatic active site, which can then cleave adjacent homodimers in the region between large (p18) and small (p10) catalytic subunits. This cleavage enables the second activation step to take place, which is intradimer cleavage in the linker region between p18 and the DEDs. The p18/p10 heterotetramers that are formed can be released from the complex and activate apoptosis by cleaving procaspases‐3/7 and BID. (B) Formation of a heterodimer between the (pseudo)catalytic regions procaspase‐8 and FLIP(L) is energetically favorable compared with caspase‐8 homodimers. This promotes formation of a FLIP(L)/caspase‐8 heterodimeric enzyme that can efficiently cleave adjacent procaspase‐8 homodimers between their large and small catalytic subunits, thereby promoting processing of these homodimers. The heterodimer also cleaves adjacent heterodimers and RIPK1. The lack of critical cysteine in FLIP(L)’s ‘active site’ and lack of a suitable target site for caspase‐8 in the region between FLIP(L)’s DED2 and p20 subunit prevent intradimer cleavage of the heterodimer, which is therefore retained in the complex.

In order to be fully processed, procaspase‐8 must form homodimers, with this dimerization initially occurring via their tandem DEDs and then via their caspase domains, which align in an antiparallel fashion to create two catalytically active sites (Fig. 2). Following homodimerization, processing occurs in two steps: interdimer processing 24, followed by intradimer processing 25 (Fig. 3A):

- Interdimer cleavage between 2 proximal dimers occurs at Asp^374^, breaking the linking region between the large and small catalytic subunits. This results in the generation of two fragments, a p43/41 subunit and a p12 subunit, although it is important to note that the cleaved small subunits from each member of the homodimer remain in the complex and are essential for forming the active site of the intermediate partially processed enzyme.
- The second stage is intradimer cleavage at either Asp^210^, Asp^216^, or Asp^223^ located between DED2 and the large catalytic subunit and at Asp^384^ in the small catalytic subunit. These intradimer cleavage events yield the apoptotically active enzyme, a heterotetramer comprised of two p10 and two p18 subunits, with two active sites 25, 26.

The first rate‐limiting step requires the proximity of two procaspase‐8 homodimers in adjacent DISCs, which is why receptor ‘superclustering’ is needed for efficient activation of apoptosis signaling 22. The active heterotetramer generates a downstream caspase signaling cascade by cleaving and activating procaspase‐3 and procaspase‐7 and the B‐cell lymphoma 2 (Bcl‐2) family member BH3‐interacting domain death agonist (BID), with the latter driving apoptosis via the intrinsic mitochondrial pathway 24, 25, 26, 27, 28, 29.

Through their tandem DEDs, all FLIP splice forms are capable of forming heterodimers with procaspase‐8 (Fig. 3B). Crucially, the FLIP(S)/(R) heterodimers with procaspase‐8 are catalytically inactive, as there is no pseudocatalytic domain to interact with the procaspase‐8 catalytic domain 30. In contrast, FLIP(L)/procaspase‐8 heterodimers have catalytic activity, with the pseudocatalytic domain of FLIP(L) interacting with the catalytic domain of procaspase‐8 in a manner similar to that in procaspase‐8 homodimers to create a membrane‐bound enzyme with a single caspase‐8 active site (Fig. 2). The FLIP(L)/procaspase‐8 heterodimeric enzyme is capable of initiating cleavage of both neighboring procaspase‐8 homodimers and other FLIP(L)/procaspase‐8 heterodimers between their large and small (pseudo)catalytic domains 31, 32, 33. Another notable substrate of the FLIP(L)/procaspase‐8 heterodimer is receptor‐interacting serine/threonine‐protein kinase 1 (RIPK1), the significance of which will be discussed below.

FLIP(L)/procaspase‐8 heterodimer‐mediated cleavage of adjacent corecruited procaspase‐8 homodimers and other FLIP(L)/procaspase‐8 heterodimers occurs between the large and small catalytic subunits in a similar manner to that described above for the homodimers (Step 1: interdimer cleavage)*.* In fact, the FLIP(L)/procaspase‐8 heterodimer is more efficient at catalyzing this first rate‐limiting activation step than the procaspase‐8 homodimer. This appears to be related to a lower activation energy for the formation of the heterodimer’s active site 34. Interdimer cleavage of FLIP(L)/procaspase‐8 heterodimers by adjacent hetero‐ and homodimers results in the generation of p43‐ and p12‐FLIP(L) cleavage products and p41/43‐ and p12‐procaspase‐8 cleavage products (Fig. 3B). As with the homodimer, the small (pseudo)‐catalytic C‐terminal cleavage products formed by interdimer cleavage of the heterodimer continue to interact with the large subunits and are essential for the catalytic activity of the heterodimeric enzyme. However, unlike the homodimer, subsequent intradimer cleavage is prohibited by the lack of catalytic activity in the FLIP(L) pseudo‐caspase domain, which cannot therefore cleave Asp^210^, Asp^216^, or Asp^223^ between DED2 and the large catalytic subunit of caspase‐8. Moreover, there is a lack of a suitable caspase‐8 cleavage site between FLIP(L)’s DED2 and its large pseudocatalytic subunit. Therefore, further processing (intradimer cleavage) of the FLIP(L)/procaspase‐8 heterodimer is prevented and the heterodimeric enzyme remains bound at the DISC, where it can cleave local substrates (adjacent dimers and RIPK1), but cannot propagate an apoptotic signal 31, 32, 33. The heterodimer also has additional functions through FLIP(L)’s interaction with other proteins at the DISC, such as TNF receptor‐associated factor 2 (TRAF2), which are described later.

### Models of DISC assembly

In recent years, a number of models of DISC assembly have been proposed 35, 36. In 2012, it was reported simultaneously by two groups that FADD and FLIP are highly substoichiometric at the DISC relative to procaspase‐8, and it was subsequently proposed that the DISC is composed of DED chains in which procaspase‐8 is the predominant protein 21, 22. In a further development, cryo‐EM studies suggested that individual DED chains interact with one another to form helical filaments 37. It was later proposed that recruitment of FLIP(S) into procaspase‐8 DED chains terminates the chains and thereby limits procaspase‐8 activation, whereas FLIP(L) promotes DED‐mediated procaspase‐8 oligomerization and thereby promotes caspase‐8 activation 35. However, an apoptosis‐promoting function for FLIP(L) is at odds with a number of siRNA‐based and overexpression studies, which indicate an anti‐apoptotic function 38, 39, 40, 41. Our recent alternative model suggests the existence of shorter procaspase‐8/FLIP tandem DED chains linking FADD molecules attached to adjacent DR trimers 42. This model can explain the ability of FLIP(L) to both activate and inhibit DISC‐mediated apoptosis 35, 43; as depending on the levels of procaspase‐8 and FLIP(L) in the short chains, there are two potential scenarios:

- Apoptosis inhibition: High levels of FLIP(L) will result in a predominance of FLIP(L)/procaspase‐8 heterodimers; therefore, the lack of procaspase‐8 homodimers will prevent sufficient processing of procaspase‐8 to its apoptosis active heterotetramer.
- Apoptosis activation: Low levels of FLIP(L) will result in a predominance of procaspase‐8 homodimers; in this scenario, the FLIP(L)/caspase‐8 heterodimer can promote the rate‐limiting first cleavage step of adjacent procaspase‐8 homodimers and thereby promote apoptosis induction.

We have recently defined the stoichiometry of FLIP(L):procaspase‐8 at the DISC at which these two opposing functions operate 42. Generally, total cellular levels of procaspase‐8 exceed those of FLIP(L) even in cancer cells which have elevated FLIP expression; however, FLIP DISC recruitment is more efficient 44. At low levels of receptor activation, the number of DISCs formed will be low and there will therefore be a predominance of heterodimers (FLIP(L):procaspase‐8 ratio ~ 1 : 1) and apoptosis will be inhibited (scenario 1). At high levels of receptor activation, FLIP(L) levels will become depleted relative to the more highly expressed procaspase‐8 (FLIP(L):procaspase‐8 ratio << 1 : 1) and there will therefore be a predominance of homodimers, the activation of which will be accelerated by corecruited heterodimers (scenario 2). Thus, FLIP(L) acts in what could be considered a classic pseudoprotease manner at the DISC, functioning to regulate the rate and extent of activation of the protease from which it evolved. The picture is much simpler for FLIP(S) and FLIP(R) as they form inactive heterodimers with procaspase‐8, and so act as straightforward inhibitors of procaspase‐8 processing and apoptosis induction 30.

### Tumor necrosis factor receptor (TNFR) complex II

Similar to its action in regulating the TRAIL‐R1/DR4, TRAIL‐R2/DR5, and Fas/CD95 receptors, FLIP(L) also plays an important role in the downstream regulation of the TNFR1. Like the aforementioned receptors, TNFR1 is a member of the TNF receptor superfamily. Upon activation by its cognate ligand TNF‐α, formation of the TNFR1 complex I occurs. Initially, the adaptor protein TNFR1‐associated death domain protein (TRADD) is recruited followed by RIPK1, TRAF2, and cellular inhibitor of apoptosis proteins‐1/2. This complex is subsequently capable of activating both nuclear factor kappa‐light‐chain‐enhancer of activated B cells (NF‐κB) and mitogen‐activated protein (MAP) kinase signaling pathways in a manner dependent on RIPK1 ubiquitination by the linear ubiquitin chain assembly complex 45, 46. Following complex I formation, RIPK1 is deubiquitinated by CYLD lysine 63 deubiquitinase, resulting in the generation of a secondary complex termed TNFR1 complex II. This is comprised of TRADD and RIPK1 dissociated from complex I, which can then recruit FADD, procaspase‐8, and FLIP in a manner akin to what occurs during DISC formation 47.

Similar to the DISC, FLIP(L), and in particular the ratio of procaspase‐8 homodimers to procaspase‐8/FLIP(L) heterodimers, plays a key role in determining signaling from TNFR1 complex II. In order for apoptosis‐inducing levels of caspase‐3/7 activity to be generated, sufficient numbers of procaspase‐8 homodimers must be recruited to the complex. In instances where procaspase‐8/c‐FLIP heterodimers are in greater abundance, this activation will be impeded. Moreover, as *CFLAR*/c‐FLIP is an NF‐κB target gene, the levels of FLIP expression are enhanced by TNFR1 complex I, which therefore acts to modulate the signaling output from complex II 48, 49.

Procaspase‐8/FLIP heterodimers formed at TNFR1 complex II can also modulate necroptosis, a pro‐inflammatory and caspase‐independent form of cell death 50, 51, 52. RIPK1 plays a central role in promoting necroptosis, phosphorylating RIPK3 which ultimately leads to the formation of the necrosome containing mixed lineage kinase domain‐like protein (MLKL). The pseudokinase MLKL is then phosphorylated itself, leading to its oligomerization and formation of pores in the plasma membrane that result in necrotic lysis of the cell, with the release of a range of pro‐inflammatory damage‐associated molecular patterns (DAMPs). As mentioned above, the procaspase‐8/FLIP(L) heterodimer can cleave RIPK1, thus blocking necroptosis 53. This is another example of FLIP(L) acting as a classic pseudoenzyme: In this instance, it functions to inhibit caspase‐8‐mediated apoptosis by complex II, but promotes cleavage of RIPK1 by its enzymatic counterpart to prevent necroptosis. FLIP(S) also inhibits apoptosis via caspase‐8, but cannot block necroptosis mediated by RIPK1 as it has no catalytic activity. Thus, both FLIP splice forms modulate the activity of caspase‐8 in these complexes, leading to distinct signaling outcomes.

## Caspase‐8‐independent roles of FLIP

**Fig. 4:**
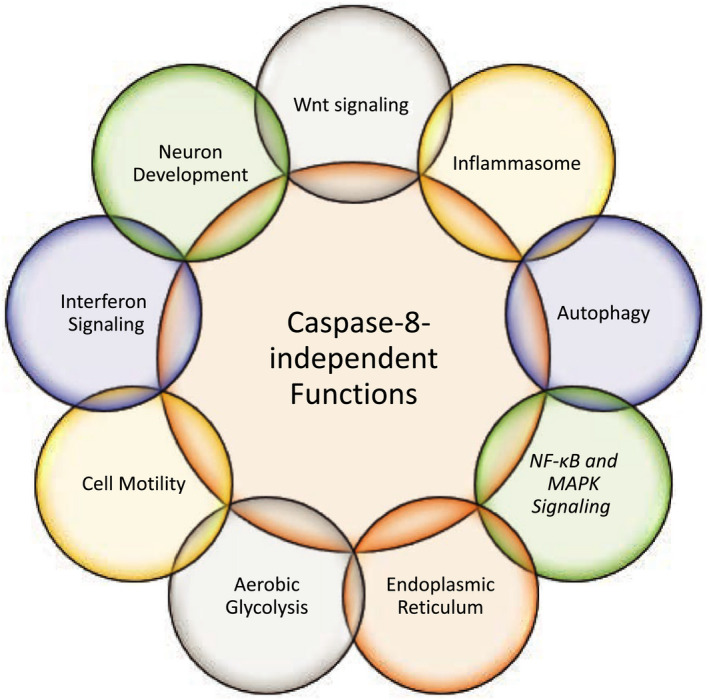
Schematic summary of some of the key caspase‐8‐independent roles of FLIP(L). In addition to functions directly related to its active paralog caspase‐8, FLIP(L) also acts independently. These nonclassical roles of FLIP(L), a selection of which are summarized, comprise an array of key regulatory functions across a wide range of biological processes, not limited to the modulation of cell death.

In addition to its roles when functioning as part of a complex with caspase‐8, FLIP(L) also has nonclassical pseudoenzyme functions that are independent of caspase‐8 (Fig. 4).

### Inflammasome

Inflammasomes are intracellular multimeric protein complexes primarily responsible for the processing of the inflammatory caspase, procaspase‐1, in response to a range of damage‐ and pattern‐associated molecular patterns (DAMPs and PAMPs) 54. Following recruitment, the inactive procaspase‐1 oligomerizes, resulting in its autoproteolytic cleavage and, in turn, its activation 54, 55. Following its activation, caspase‐1 in turn activates interleukin (IL)‐1β and IL‐18 through cleavage of their precursor cytokines (pro‐IL‐1β and pro‐IL‐18) 56. Active caspase‐1 has also been shown to have a role in the induction of pyroptosis (an inflammatory form of immune cell death) through the cleavage of gasdermin D 57, 58.

Inflammasomes are classified based on the protein upon which their scaffold structure is formed 59. One of the most important and best understood is the NACHT, LRR, and pyrin domain (PYD)‐containing protein 3 (NLRP3) inflammasome, which, compared to other inflammasomes, is triggered in response to the most diverse range of stimuli 60. Activation of caspase‐1 via this inflammasome occurs in what could be considered a two‐step process 61, 62. Classically, activation of NF‐κB in response to DAMP/pathogen‐associated molecular pattern (PAMP)‐mediated activation of toll‐like receptors (TLRs) upregulates expression of the IL precursor cytokines (pro‐IL‐1β and pro‐IL‐18) and NLRP3 itself 63. This is followed by the formation of the inflammasome complex, comprising NLRP3, procaspase‐1, and the adaptor protein apoptosis‐associated speck‐like protein containing CARD (ASC), at which procaspase‐1 oligomerization results in its activation with subsequent cleavage and activation of pro‐IL‐1β and pro‐IL‐18 64.

FLIP(L) has been shown to directly influence the NLRP3 inflammasome: Through its pseudo‐caspase domain, FLIP(L) is capable of interacting directly with both NLRP3 and procaspase‐1, thereby modulating the activity of the inflammasome 65. The interaction of FLIP(L) with the NLRP3 inflammasome leads to an increase in the secretion of IL‐1β, while its absence has been implicated in the inhibition of pyroptosis. In addition to its interaction with and modulation of the NLRP3 inflammasome, FLIP(L) can also modulate the interferon‐inducible protein AIM2 (AIM2) inflammasome, which is comprised of the absent in melanoma 2 (AIM2) protein scaffold. AIM2 contains an N‐terminal PYD and a hematopoietic interferon‐inducible nuclear antigen (HIN) domain, located at the C terminus 66. The AIM2 inflammasome is activated by cytosolic DNA, from either the host or foreign microbial DNA. Following activation of AIM2, procaspase‐1 is recruited. Similar to the NLRP3 inflammasome, this recruitment is reliant on the adaptor protein ASC. Following recruitment, autoproteolytic activation yields activated caspase‐1 that in turn processes pro‐IL‐1β and pro‐IL‐18 and cleaves gasdermin D 66. It has been shown that the caspase‐like domain of FLIP(L) is capable of binding directly to the C‐terminal HIN region of AIM2 65. In turn, this interaction was shown to be required to achieve full AIM2 activation.

Although procaspase‐8 has also been shown to modulate the inflammasome, these functions, which are specific for the long FLIP splice form, are not reported to be caspase‐8‐dependent and are therefore examples of FLIP(L) acting in a nonclassic pseudoenzyme manner 67.

### Wingless‐related integration site (Wnt) signaling

Independently of procaspase‐8, FLIP(L) has also been implicated in playing a role in the Wnt signaling pathway. This signaling cascade is induced following the binding of Wnt proteins to the N‐terminal region of Frizzled receptors 68, 69. In the absence of receptor binding, degradation of β‐catenin is mediated by the ‘destruction complex’ formed by adenomatous polyposis coli (APC), Axin, casein kinase 1, and glycogen synthase kinase 3 beta, in which β‐catenin is phosphorylated and then targeted for proteasomal degradation 70. Following Frizzled receptor binding by Wnt, a coreceptor complex is formed with LRP5/6 71. This membrane complex binds Axin, preventing formation of the destruction complex, leading to stabilization of β‐catenin, which then translocates to the nuclear compartment where it cooperates with the T‐cell factor/lymphoid enhancer‐binding factor transcription factors to drive expression of various genes that regulate stemness and proliferation 72. This pathway is particularly important in the large intestine, with its disruption through mutation/loss of the *APC*/APC gene or amplification of β‐catenin being an initiating event in the majority of colorectal cancers 73, 74, 75, 76.

FLIP(L) has been reported to inhibit β‐catenin ubiquitination, leading to increases in cytosolic β‐catenin and its translocation to the nucleus, where it promotes activation of its target genes 77. The importance of FLIP to Wnt signaling was further illustrated through FLIP depletion, which caused a reduction in Wnt signaling. It was also demonstrated that this action of FLIP was FADD‐dependent. Interestingly, however, procaspase‐8 appeared to have no discernible role in regulating this interaction. One point of note regarding this function of FLIP(L) is the importance of its cellular localization in mediating this effect 78. FLIP(L) has both a nuclear localization signal and a nuclear export signal (NES) within its C terminus (not present in the other FLIP splice forms). These features allow the accumulation of FLIP(L) in both cytoplasmic and nuclear compartments. Mutation of FLIP(L)’s nuclear localization sequence (NLS) constricted its localization to the cytoplasm and was shown to inhibit FLIP(L)’s effects on Wnt signaling.

More recently, it has been reported that FLIP(L) could interact via its DED with the nuclear protein TBP‐interacting protein 49 (TIP49) and that this interaction was crucial for the ability of FLIP(L) to modulate Wnt signaling 79. In this study, FLIP(L) was shown to mediate the accumulation of TIP49 at the immunoglobulin transcription factor‐2 (ITF‐2) promoter, which is itself targeted by β‐catenin. The localization of TIP49 at the ITF‐2 promoter was shown to be crucial for its regulation of β‐catenin–TCF transcriptional complex.

### Autophagy

Another process in which the pseudo‐caspase FLIP(L) has a role to play is in autophagy. Interestingly, in modulating this cellular function, FLIP(L) can act both independently or in concert with its other genetic paralog, procaspase‐10.

Autophagy is a degradation process in which cytoplasmic proteins are packaged into autophagosomes that in turn are delivered to lysosomes, where an autolysosome is formed, and the packaged cytoplasmic material degraded 80. Material that is degraded in this manner includes a variety of unrequired long‐lived proteins and dysfunctional complexes and organelles 81. Autophagy is induced in instances of cellular stress and, while normally tightly controlled, its deregulation is apparent in a number of pathologies 80. Autophagosome formation is reliant upon a group of proteins termed autophagy‐related proteins (Atg). One such Atg, Atg3, has been shown to play an important role in binding and subsequent processing of microtubule‐associated protein 1A/1B‐light chain 3 (LC3), which is then incorporated as an essential component of the autophagosome lipid membrane 82, 83, 84.

FLIP(L) has been shown to directly bind Atg3 and impede autophagy by preventing binding of Atg3 to LC3. This in turn prevents the formation of autophagosomes 85. In addition to this direct modulation of autophagy, FLIP(L) has been shown to affect autophagy through its interaction with procaspase‐10. Beclin‐1 plays a role in autophagosome formation by inducing the localization of autophagy proteins to the pre‐autophagosomal membrane. In order to carry out this role, Beclin‐1 must be released from the anti‐apoptotic protein, Bcl‐2 86. In multiple myeloma cells, this activation of Beclin‐1 is impeded by the presence of FLIP(L): DED‐mediated heterodimerization of procaspase‐10 and FLIP(L) results in a complex possessing proteolytic activity that cleaves the BCL2‐interacting protein Bcl‐2 associated transcription factor 1 (BCLAF1). This inhibits the ability of BCLAF1 to activate Beclin‐1 by displacing Bcl‐2, in turn preventing Beclin‐1‐mediated autophagy. Therefore in this setting, upon heterodimerization with procaspase‐10, FLIP(L) functions as a negative regulator of Beclin‐1‐mediated autophagy 87. In both cases (direct interaction with Atg3 and cleavage of BCLAF1), FLIP(L) acts independently of caspase‐8; however, the involvement of caspase‐10 in the latter scenario means that this is a classic pseudoprotease function (modulating the activity of an enzymatically active paralog).

### NF‐κB and MAPK signaling

FLIP(L) has also been reported to play a role in the modulation of NF‐κB signaling. It has been reported that FLIP(L), recruited following activation of the Fas DISC, was capable of interacting with both TRAF‐1 and TRAF‐2, as well as with kinases RIPK1 and RAF proto‐oncogene serine/threonine‐protein kinase (Raf‐1) 88. This had an activating effect on both NF‐κB and extracellular signal‐regulated kinase (ERK) signaling. Interestingly, when considering FLIP(L) as a pseudoprotease, the interactions between it and both TRAF1 and RIPK1 were reported to be predominantly mediated by the FLIP(L) pseudo‐caspase domain 88. This direct NF‐κB activation by c‐FLIP has more recently been supported by work wherein the enforced expression of c‐FLIP in monocytes was also shown to activate the NF‐κB pathway and result in enhanced immunosuppressive activity 89.

Additionally, FLIP(L) has been demonstrated to be involved in the regulation and activation of T cells 90, 91. Increased FLIP(L) expression results in enhanced caspase‐8 activation and a concomitant increase in T‐cell proliferation. This has been suggested to result from FLIP(L) functioning as a caspase‐8 activator and substrate, enhancing NF‐κB activation. It was demonstrated in this setting that cleavage of FLIP(L) at Asp^376^, yielding p43‐FLIP, led to enhanced/more effective RIPK1 recruitment. This activation of caspase‐8 and cleavage of FLIP(L) was further implied to be associated with migration of FLIP(L) and caspase‐8 to lipid rafts 92. This localization to lipid raft regions has also been noted for other protein complexes required for TCR‐mediated NF‐κB activation 93, 94.

An alternative mechanism of FLIP regulation of NF‐κB signaling was reported in which p22‐FLIP, a nonclassical cleavage product formed by cytoplasmic c‐FLIP and procaspase‐8 heterodimers, directly binds to and modulates the activity of the IκB kinase (IKK) complex, a critical upstream of regulator of the NF‐κB signaling pathway 95. In these DED‐mediated, apparently FADD‐independent heterodimers, procaspase‐8 can cleave both FLIP(L) and FLIP(S) at Asp198 to generate p22‐FLIP, an amino‐terminal fragment containing DED1 and DED2. Specifically, p22‐FLIP (as well as viral forms of FLIP) binds NEMO/IKKγ, the regulatory component of the IKK complex, strongly activating complex activity and resulting in potent NF‐κB activity.

As noted above, following ligation of TNFR1 by TNF‐α, NF‐κB activation by complex I causes upregulation of FLIP(L) expression levels. FLIP(L) was subsequently shown to be essential for TNF‐α‐induced MAPK activation, mediating these effects through its interactions with Raf‐1 in a Ras‐independent manner 96. Furthermore, it has been found that FLIP(L) directly interacts with the c‐Jun N‐terminal kinase (JNK) activator MAP kinase kinase 7 (MKK7), following TNF‐α stimulation 97. This in turn results in the inhibition of interactions involving MKK7 with MAP/ERK kinase kinase 1 (MEKK1), apoptosis signal‐regulating kinase 1, and TGF‐β‐activated kinase 1. This has the effect of preventing JNK‐dependent reactive oxygen species accumulation. The caspase‐like domain was again noted to be essential for FLIP(L) binding to MKK7, with residues 261–327 necessary for inhibition of the MKK7–JNK pathway.

### Endoplasmic reticulum

It has been reported that FLIP(L) plays a role in both determining ER morphology and modulating the functions of ER mitochondria‐associated membranes 98. In models of FLIP ablation, the ER displayed notable morphological abnormalities in addition to the uncoupling of the ER from mitochondria. Furthermore, lack of FLIP resulted in increased cleavage of Reticulon‐4 by caspase‐7. Moreover, recent investigations have indicated that loss of FLIP(L) results in a protein kinase B (AKT) activation in response to ER stress, leading to inhibition of protein kinase RNA‐like endoplasmic reticulum kinase (PERK) and inositol‐requiring enzyme 1 signaling; this suggests a further role for FLIP(L), through modulating AKT activity and PERK signaling, in mediating the cellular response to ER stress 99.

### Aerobic glycolysis

Using hepatocellular carcinoma cells, it was recently reported that when FLIP(L) was overexpressed, aerobic glycolysis was accelerated 100. This was demonstrated by increases in glucose consumption, uptake, and production of the by‐product lactate. Furthermore, this effect of FLIP(L) was found to be related to the sodium/glucose cotransporter 1 (SGLT1), with high levels of FLIP(L) expression correlated with high levels of SGLT1. Notably, it was found that FLIP(L) was able to bind SGLT1, with this interaction inhibiting SGLT1 ubiquitination and proteasome‐mediated degradation, thereby promoting glucose transport.

### Cell motility

It has been reported that in HeLa cells, FLIP(L) was capable of enhancing motility and also adhesion to extracellular matrix proteins 101. Interestingly, FLIP(S) did not elicit these same effects. Mechanistically, FLIP(L) was found to induce motility through the Rho‐associated signaling pathway, leading to focal adhesion kinase activation, which in turn activated ERK. In addition, FLIP(L) increased the expression of matrix metallopeptidase 9 which also promotes increased cell motility. Amino acids 222 and 376 in FLIP(L)’s pseudo‐caspase domain were found to mediate these effects.

### Interferon signaling

Interferon (IFN) regulatory transcription factor 3 (IRF3) is a transcription factor that promotes expression of IFNα, IFN β, and other interferon‐stimulated genes, which in turn moderate cellular responses to, for instance, viral infection 102. FLIP(L) was found to function as an inhibitor of IRF3‐induced gene expression 103. This activity was found to be a result of FLIP(L) preventing of IRF3’s interaction with CREB‐binding protein. This is a particularly interesting mode‐of‐action as unlike other IRF3 regulators, it does not rely on the ability of the inhibiting molecule (in this case FLIP(L)) to either dephosphorylate or degrade IRF3. Also of note was the finding that this inhibitory effect is dependent on the caspase‐like domain of FLIP(L), which was found to be capable of mediating these effects in the absence of its DEDs.

### Neuron development

In an embryonic mouse model, it was observed that FLIP(L) was significantly expressed in neurons, with these levels subsiding later in development 104. Neuronal development is modulated by neurotrophins, such as nerve growth factor (NGF) and brain‐derived neurotrophic factor 105. These neurotrophins can bind to the Trk receptor tyrosine kinase and the p75 neurotrophin receptor, thereby impacting both neuronal survival and differentiation 106, 107, 108, 109. It has been suggested that FLIP(L) overexpression promotes neurotrophin‐induced neurite outgrowth 104. Indeed, even in the setting of sufficient neurotrophin levels, reduction in FLIP(L) significantly impeded neurite growth. These effects of FLIP(L) on neuritogenesis were dependent on signaling from both the MAPK/ERK and NF‐κB pathways. Additionally, FLIP(L) was shown to be capable of directly binding the neurotrophin receptor TrkA, in a NGF‐dependent manner.

## Concluding remarks

To our knowledge, FLIP(L) is the only true pseudoenzyme of the caspase enzyme family in humans. In the majority of humans, the *CASP12* gene encodes a truncated protein due to premature stop codon, so it is probably more correctly classified as a pseudo*gene* 110. In addition to its canonical functions as a classical pseudoenzyme, FLIP(L) has evolved a significant number of additional functions, potentially fueled by its high rate of evolution. Thus, FLIP(L) has been shown to modulate a range of processes including inflammation, motility, and proliferation. While disparate in nature, it is notable that these nonclassical pseudoenzyme functions are, like its canonical functions, also associated with the development and progression of cancer.

## Conflict of interest

The authors declare no conflict of interest.

## Author contributions

PS and TS wrote the manuscript and prepared figures. CJS and DBL edited and revised the manuscript.
